# Molecular Mechanisms and Biological Functions of siRNA

**Published:** 2017-06

**Authors:** Hassan Dana, Ghanbar Mahmoodi Chalbatani, Habibollah Mahmoodzadeh, Rezvan Karimloo, Omid Rezaiean, Amirreza Moradzadeh, Narges Mehmandoost, Fateme Moazzen, Ali Mazraeh, Vahid Marmari, Mohammad Ebrahimi, Mohammad Menati Rashno, Saeid Jan Abadi, Elahe Gharagouzlo

**Affiliations:** 1 Department of Biology, Damghan Branch, Islamic Azad University, Damghan, Iran;; 2 Cancer Research Center, Cancer Institute of Iran, Tehran University of Medical Science, Tehran, Iran;; 3 Department of Medicine, Zahedan Medical Science, Zahedan, Iran;; 4 Department of chemistry, University of Sistan and Baluchestan, Zahedan, Iran;; 5 Department of Laboratory sciences, Zahedan Branch, Islamic Azad University, Zahedan, Iran;; 6 Department of Biology, University of Technology, Hefei, China;; 7 Department of Microbiology, Shiraz Medical Science, Shiraz, Iran

**Keywords:** siRNA, Delivering siRNA, RNAi, Gene silencing, siRNA therapeutics

## Abstract

One of the most important advances in biology has been the discovery that siRNA (small interfering RNA) is able to regulate the expression of genes, by a phenomenon known as RNAi (RNA interference). The discovery of RNAi, first in plants and *Caenorhabditis elegans* and later in mammalian cells, led to the emergence of a transformative view in biomedical research. siRNA has gained attention as a potential therapeutic reagent due to its ability to inhibit specific genes in many genetic diseases. siRNAs can be used as tools to study single gene function both in vivo and in-vitro and are an attractive new class of therapeutics, especially against undruggable targets for the treatment of cancer and other diseases. The siRNA delivery systems are categorized as non-viral and viral delivery systems. The non-viral delivery system includes polymers; Lipids; peptides etc. are the widely studied delivery systems for siRNA. Effective pharmacological use of siRNA requires ‘carriers’ that can deliver the siRNA to its intended site of action. The carriers assemble the siRNA into supramolecular complexes that display functional properties during the delivery process.

## INTRODUCTION

In recent years, few areas of biology have been transformed as thoroughly as RNA molecular biology. This transformation has occurred along many fronts, as detailed in this issue, but one of the most significant advances has been the discovery of small (20–30 nucleotide [nt]) noncoding RNAs that regulate genes and genomes. This regulation can occur at some of the most important levels of genome function, including RNA processing, chromatin structure, RNA stability, chromosome segregation, transcription, and translation (1-3).

Despite many classes of small RNAs have emerged, biological roles, associated effector proteins, various aspects of their origins, and structures have led to the general recognition of three main categories: piwi-interacting RNAs (piRNAs), short interfering RNAs (siRNAs), and microRNAs (miRNAs) (4-7).

RNA interference (RNAi), the biological mechanism by which double-stranded RNA (dsRNA) induces gene silencing by targeting complementary mRNA for degradation, is a tremendous innovation in the universal therapeutic treatment of disease and revolutionizing the way researchers study gene function. RNAi, first discovered in plants, was later demonstrated in the roundworm Caenorhabditis elegans, an organism in which gene expression is downregulated by long dsRNA (8, 9).

Increasing knowledge on the molecular mechanisms of endogenous RNA interference, siRNAs have been emerging as innovative nucleic acid medicines fo the treatment of incurable diseases such as cancers (10-14). Because systemic administration will be required in most cases, there are challenges inherent in the further development of siRNAs for anti-cancer therapeutics, Although several siRNA candidates for the treatment of respiratory and ocular diseases are undergoing clinical trials (15-17).

In an attempt to develop siRNA for use in clinical trials as drugs, various chemical modifications are being investigated to improve qualities such as low immunostimulation, target organ/cell delivery, off-target effects, siRNA potency, and serum stability [18].

Despite the high therapeutic potential of siRNA, its application in clinical settings is still limited mainly due to the lack of efficient delivery systems [19-23].

The discovery of RNAi has opened doors that might introduce a novel therapeutic tool to the clinical setting [24-32]. RNAi is charged with controlling vital processes such as cell growth, tissue differentiation, heterochromatin formation, and cell proliferation. Accordingly, RNAi dysfunction is linked to cardiovascular disease, neurological disorders, and many types of cancer [33]. RNAi pathways transcend mere expansion of the gene regulation toolkit: They confer a qualitative change in the way cellular networks are managed [34].

## MECHANISM OF RNA INTERFERENCE (RNAI)

**Figure 1:**
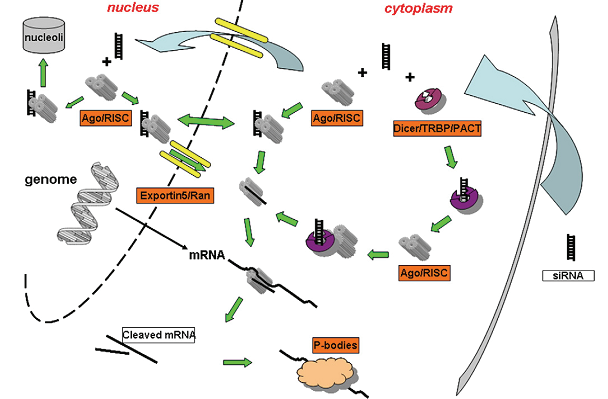
Schematic of the siRNA mediated RNA interference pathway. After entry into the cytoplasm, siRNA is either loaded onto RISC directly or utilize a Dicer mediated process. After RISC loading, the passenger strand departs, thereby commencing the RNA interference process via target mRNA cleavage and degradation. siRNA loaded RISCs are also found to be associated with nucleolus region and maybe shuttled in and out of nucleus through an yet unidentified process [48].

The first step of RNAi involves processing and cleavage of longer double-stranded RNA into siRNAs, generally bearing a 2 nucleotide overhang on the 3′ end of each strand. The enzyme responsible for this processing is an RNase III-like enzyme termed Dicer [35-38]. When formed, siRNAs are bound by a multiprotein component complex referred to as RISC (RNAinduced silencing complex) [39-42]. Within the RISC complex, siRNA strands are separated and the strand with the more stable 5′-end is typically integrated to the active RISC complex. The antisense single-stranded siRNA component then guides and aligns the RISC complex on the target mRNA and through the action of catalytic RISC protein, a member of the argonaute family (Ago2), mRNA is cleaved [43-47] (Figure 1).

## ORIGINS siRNAs

Fire and Mello In 1998 uncovered the world of RNAi and revolutionized the contemporary understanding of gene regulation when they made the discovery that the silencing effectors in Caenorh abditis elegans were double stranded RNAs [49].

In 1999, siRNAs were discovered in plants and similarly demonstrated to guide sequence-dependent endonucleolytic cleavage of the mRNAs that they regulate in mammalian cells [50]. By 2001, miRNAs were found to comprise a broad class of small RNA regulators, with at least dozens of representatives in each of several animal and plant species [51]. With this discovery, in our view of the gene regulatory landscape two categories of small RNAs had become firmly embedded: siRNAs, as defenders of genome integrity in response to foreign or invasive nucleic acids such as transposons, transgenes, and viruses, and miRNAs, as regulators of endogenous genes [52].

Elbashir et al. in 2001 had successfully used synthetic siRNAs for silencing and determined the basic principles of siRNA structure and RNAi mechanics, providing the foundation for developing RNAi applications [53].

## CHALLENGES WITH siRNA-BASED THERAPEUTICS

### Delivering siRNA

The discovery of RNAi has excited the scientific field due to its potential for wide range of therapeutic applications [54-58]. Effective pharmacological use of siRNA requires ‘carriers’ that can deliver the siRNA to its intended site of action. The carriers assemble the siRNA into supramolecular complexes that display functional properties during the delivery process [59]. Viral vectors and non-viral vectors are two major categories delivery system for siRNA. The synthesis and industrial scalability are offer advantages of Non-viral delivery systems. Peptides, polymers, and Lipids are the extensively studied non-viral delivery vehicles [60-70].

The pharmacological mediator of siRNA, has faced significant obstacles in reaching its target site and effectively exerting its silencing activity.

The fantastic potential of siRNA to silence important genes in disease pathways comes with noteworthy challenges and barriers in its delivery.

### Polymer- mediated Delivery Systems

For siRNA delivery, Polymers have emerged as an alternative class of extensively investigated carriers [71, 72]. Polymer-based delivery systems have been extensively used for plasmid DNA and more recently for siRNA [73-75]. As non-viral siRNA and plasmid vectors, many polymers have been thoroughly investigated because of their physical characteristics and diverse chemistries and well-characterized, and structure flexibilities, which allows for easy modification to fine-tune their physiochemical properties. Chitosan is reported to have low cytotoxicity and which is a cationic polysaccharide having muco adhesive properties. Cyclodextrin is another polymer that has also been studied as siRNA delivery system [76-88].

### Peptide-Based Delivery Systems

Due to four major reasons, Peptides are viewed as alternative to the cationic polymers for the siRNA delivery: Cell specific delivery, pH based membrane, disruption, Their efficient packaging and Efficient membrane transport. Control over functionalization, ease of synthesis and stability of the peptide oligonucleotide complex make the low molecular weight peptides as the favorable candidates over lipoplexes as siRNA delivery vehicle [89-100].

Cell penetrating peptides (CPPs), also referred to as protein transduction domains (PTD), was observed to cross the plasma membrane by itself, which transactivates transcription of the HIV-1 genome, were first discovered a few decades ago when the HIV-1 Tat-protein. CPPs are the widely studied peptides as siRNA delivery system. The CPPs in three classes are classified: 1) synthetic ones, 2) chimeric peptides and 3) naturally derived peptides [101-106]. MPPs (Membrane perturbing peptides) are also studied as peptide delivery systems. Depending upon the DNA release behavior, The MPPs are studied in two categories: Endoosmolytic peptides and Fusogenic peptides that Fusogenic peptides act by mediating the DNA release at the endosomal pH and Endoosmolytic peptides act by endosomal lysis followed by DNA release [107].

### Lipid -Based Delivery Systems

Various lipid-based delivery systems have been developed for in vivo application of siRNA. Lipid-based systems include liposomes, micelles, emulsions, and solid lipid nanoparticles [108-121].

MIT(Massachusetts Institute of Technology) and Alnylam Inc., (Cambridge, Massachusetts) collaborated on a project to synthesize a library of more than thousand different lipid-like molecules and screened them for their efficiency to deliver siRNA [122]. They tested these delivery systems in mice to treat the respiratory ailment and found that some of their molecules are ten times more efficacious in delivering siRNA in comparison to the existing non encapsulated siRNA delivery.

For the delivery of siRNA using lipid-based systems, particle size, lipid composition, drug-to lipid ratio, and the manufacturing process should be optimized.

**Figure 2:**
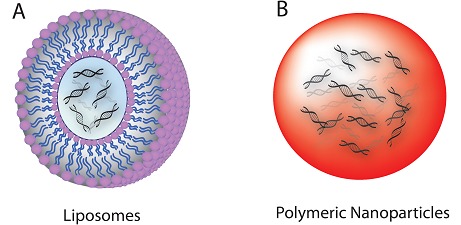
Schematic of siRna nanocarriers. a) Liposomes. B) Polymeric nanoparticles [125].

Liposomes have been utilized as efficient delivery vectors for siRNA for almost 30 years since the successful use of lipofection in 1987 to transfer nucleic acids into animal and human cells [123, 124]. Liposomes are commonly used as delivery vehicles for a broad spectrum of therapeutics including siRNA. Interaction of the lipids with the nucleic acid leads to the formation of either coated vesicles having nucleic acid in the core or the aggregates, both of which are studied as lipoplexes (Figure 2).

### siRNA Conjugate Delivery Systems

Combination of two or more types of delivery vehicles have led to the generation of this category. These combinations may be categorized as Peptide-Polymer, Liposome - Peptide, and Liposome-Polymer any other combination thereof [126-130].

Polymer and Liposomal-based delivery systems have been advanced the most for siRNA delivery, and have a vast supporting body of literature due to their extensive previous development for the delivery of antisense oligonucleotides, DNAzymes and plasmid DNA.

For targeted delivery of siRNAs to hepatocytes, Rozema et al. have developed a polymer-siRNA conjugate delivery system termed Dynamic PolyConjugates (DPCs) [131].

Recently, siRNA conjugates have shown promise as delivery platforms, leading to the development of well-defined, single-component systems that optimize the usage of minimal amounts of delivery material [131-134]. Studies have demonstrated invitro effectiveness of LSPCs (Liposome-siRNA-peptide complexes) in delivering PrP siRNA specifically to AchR-expressing cells, leading to suppressed PrPC expression and eliminated PrPRES formation [70].

### Delivery of therapeutic siRNA in cancer

The siRNA has provided new opportunities for the development of innovative medicine to treat previously incurable diseases such as cancer [135-138]. It is of inherent potency because it exploits the endogenous RNAi pathway, allows specific reduction of disease associated genes, and is applicable to any gene with a complementary sequence [139]. For the rationale of siRNA-mediated gene therapy, genetic nature of cancer provides solid support. In fact, a number of siRNAs have been designed to target dominant oncogenes, viral oncogenes involved in carcinogenesis, or mal functionally regulated oncogenes. In addition, therapeutic siRNAs have been investigated for silencing target molecules crucial for tumor–host interactions and tumor resistance to chemo- or radiotherapy. The silencing of critical cancer-associated target proteins by siRNAs has resulted in significant antiproliferative and/or apoptotic effects [140]. However, most approaches to RNAi-mediated gene silencing for cancer therapy have been with cell cultures in the laboratory, and key impediments in the transition to the bedside due to delivery considerations still remain. Delivery systems that can improve siRNA stability and cancer cell-specificity need to be developed, nonspecific immune stimulatory effects and involve the minimizing of off-target. The delivery systems must be optimized for specific cancers, as the route of administration may differ depending on the nature of cancer. The current progress in siRNA delivery systems for liver [141], Breast [142, 143], prostate [144], and lung [145,146] cancers is discussed (Table 1).

**Table 1:** Examples of siRNA delivery systems in treatment of cancers

Delivery systems Targeted gene Property Animal model			

Polymer	Her-2	PEI	Ovarian cancer xenograft (147)
Polymer	PTN	PEI	Orthotopic glioblastoma (148)
Polymer	Akt1	Poly (ester amine)	Urethane-induced lung cancer (149)
Liposome	Bcl-2	Cationic liposome	Liver metastasis mouse model (150)
Liposome	Raf-1	Cationic cardiolipin liposome	Prostate cancer xenograft (11)
Liposome	EphA2	Neutral liposomes (DOPC)	Ovarian cancer xenograft (12)
Liposome	EGFR	Liposome-polycation-DNA	Lung cancer xenograft (151)
Liposome	Her-2	Immunoliposome	Breast cancer xenograft (152)
Liposome	HBV	SNALP	HBV vector-based mouse (153)

PEI, polyethyleneimine; SNALPs, stable nucleic acid–lipid particles.

### Efficacy

In the recent years, a number of siRNAs have been successfully used in experimental models. Data from preclinical models are now giving rise to the translation of new siRNA-based therapies into clinical trials. The target selection process is extensional, requiring a thorough mining of pathways and databases [154]. Different siRNAs targeting different parts of the same mRNA sequence have varying RNAi efficacies, and only a limited fraction of siRNAs has been shown to be functional in mammalian cells [155]. Among randomly selected siRNAs, 58–78% were observed to induce silencing with greater than 50% efficiency and only 11–18% induced 90–95% silencing [156].

### Off-target silencing effect

**Figure 3:**
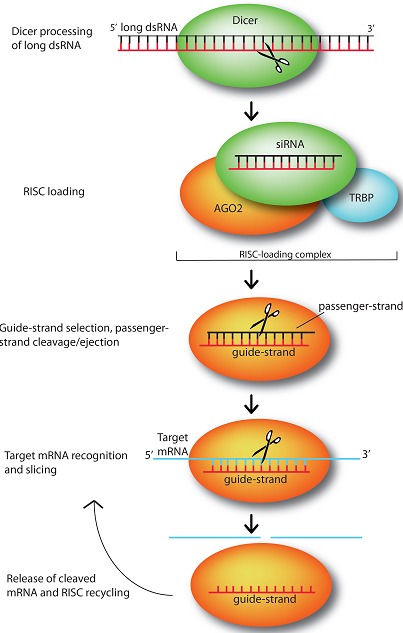
siRNA mediate silencing of target genes by guiding sequence dependent slicing of their target mRNAs. These non-coding, silencing RNAs begin as long dsRNA molecules, which are processed by endonuclease Dicer into short, active ~21-25 nt constructs. Once generated, a siRNA duplex is loaded by Dicer, with the help of RNA-binding protein TRBP, onto Argonaute (AGO2), the heart of the RNA-induced silencing complex (which here is represented just by AGO2). upon loading, AGO2 selects the siRNA guide strand, then cleaves and ejects the passenger strand. While tethered to AGO2, the guide strand subsequently pairs with its complementary target mRNAs long enough for AGO2 to slice the target. After slicing, the cleaved target mRNA is released and RISC is recycled, using the same loaded guide strand for another few rounds of slicing [161]

An early glimpse into the possible existence of off-target gene regulation by siRNAs came from gene expression profiling of siRNA studies. The specificity of RNAi is not as robust as it was initially thought to be. It is now well established that siRNA off-targets exist for many siRNA and that most siRNA molecules are probably not as specific as once thought. The introduction of siRNA can result in off-target effect, i.e. the suppression of genes other than the desired gene target, leading to dangerous mutations of gene expression and unexpected consequences [157]. The majority of the off-target gene silencing of siRNA is due to the partial sequence homology, especially within the 3’untranslated region (3’UTR), exists with mRNAs other than the intended target mRNA [158]. This mechanism is similar to the microRNA (miRNA) gene silencing effect. The off-target effect can also be a result of the immune response. RNA is recognized by immunoreceptors such as TLRs (Toll-like receptors) [159], leading to the release of cytokines and changes in gene expression. Although the sequence dependence of the immune response is not fully understood, some immunostimulatory motifs have been identified and they should be avoided [160]. Chemical modification of siRNA, such as 2’-O-methylation of the lead siRNA strand can also taper the miRNA-like off-target effects as well as the immunostimulatory activity without losing silencing effect of the target gene. Overall, therapeutic siRNA must be carefully designed. A combination of computer algorithms and empirica testing is also encouraged to allow effective design of potent siRNA sequences and minimize off-target effect (Figure 3).

## CONCLUSION

The design and engineering of siRNA carriers gained significant momentum in recent years, as a result of accumulation of predictable and therapeutically promising molecular targets. RNAi technology has progressed rapidly from an academic discovery to a potential new class of treatment for human disease. Initial observations that were useful for studying gene function in worms were quickly translated to other organisms, and in particular to mammals, revealing the potential clinical applications of siRNA, including an ability to induce persistent, potent, and specific silencing of a wide range of genetic targets. For any new therapeutics, safety is still the primary concern. While the off-target effect of siRNA is a major issue that needs to be addressed by improving the knowledge in this area, the long-term safety of siRNA is still not clear.

siRNA therapeutics are now well poised to enter the clinical formulary as a new class of drugs in the near future. siRNA-based therapies are emerging as a promising new anticancer approach, and a small number of Phase I clinical trials involving patients with solid tumours have now been completed. siRNA-based therapeutics hold great potential for cancer therapy and treatment of other diseases. However, many challenges, including rapid degradation, poor cellular uptake, and off-target effects, need to be addressed in order to carry these molecules into clinical trials. siRNA therapeutics have several distinct advantages over traditional pharmaceutical drugs. RNAi is an endogenous biological process, so almost all genes can be potently suppressed by siRNA. The identification of highly selective and inhibitory sequences is much faster than the discovery of new chemicals, and it is relatively easy to synthesize and manufacture siRNA on a large scale. As the treatment of cancers usually requires systemic delivery rather than more easily achievable local delivery, the progress of siRNA treatment for cancer has been relatively slow compared with that of other diseases curable by local siRNA administration.

Delivery, especially systemically administered siRNA, is another important barrier to be overcome. Although new materials and delivery systems are being investigated to enhance the delivery efficiency, approval procedures could be hindered by the complicated formulation. On the other hand, eyes and lungs are promising tissues for local delivery of naked siRNA, especially the former, which is reflected by the high number of clinical trial studies targeting this site. It is not surprising to see the first siRNA therapeutics to be approved is for ocular therapy in the very near future.
